# Metals in Occluded Water: A New Perspective for Pollution in Drinking Water Distribution Systems

**DOI:** 10.3390/ijerph16162849

**Published:** 2019-08-09

**Authors:** Huiyan Tong, Zhongyue Li, Xingshuai Hu, Weiping Xu, Zhengkun Li

**Affiliations:** Dalian University of Technology, No.2 Dagong Road, New District of Liaodong Bay, Panjin 124221, Liaoning, China

**Keywords:** occluded water, drinking water pollution, corrosion, cast iron, metal

## Abstract

Occluded water is water that remains inside corrosion scales within deteriorating distribution pipes. The accumulation of iron and manganese in the occluded water is a potential risk for water quality. Thus, this study investigated the change in metal (iron, manganese, copper and chromium) concentration in occluded water, the effect of these metals on the flowing water, and the source of iron and manganese in the occluded water using a simulation device. The results showed that total iron and total manganese were enriched in the occluded water, while the concentrations of total copper and total chromium in the occluded water gradually decreased over time. The iron and manganese in the occluded water migrate to the flowing water causing pollution in the flowing water. Also, copper and chromium adsorb on the corrosion scales within the pipes. The iron and manganese in the occluded water mainly came from the corrosion of the metal pipes, and the corrosion scales had a certain obstructive effect on the outward migration of iron in the occluded water but had less hindrance to the migration of manganese. Occluded water plays a critical role in the pollution of drinking water, and additional work is needed to control metal accumulation and release.

## 1. Introduction

Safe drinking water distribution issues are basic issues related to human health. Deterioration of water distribution pipes affect water quality resulting in changes such as “red water” or “black water”, which often cause customer complaints [1,2]. Water supply pipes are one of the most important facilities in urban drinking water distribution systems [3]. Compared with the problem of water source and the quality of water supplied by the factory, the urban water supply network has become a weak point of water supply security due to its wide coverage, complex system and strong randomness of water usage.

Globally, the total number of reported outbreaks of waterborne diseases has decreased since 1980, but the number of aquatic disease outbreaks associated with water system problems is on the rise [4,5]. Although drinking water from water treatment plants may meet the required standard, the final water used by customers may still be contaminated due to the deterioration of the water in the drinking water distribution systems. The final water may contain increased iron and manganese concentrations, increased turbidity, or may change in color and may even contain high numbers of bacteria [6,7,8]. The main reason for deterioration of water quality is that drinking water passes through long pipes during its distribution and has a long hydraulic retention time [2,9]. In the 1990s, water supply pipelines mainly included cold-galvanized pipes, gray-cast iron pipes and ductile iron pipes. After years of use, metal pipes have corroded in varying degrees, and most of the pipes have reached their service life [10]. However, due to water quality and hydraulic conditions, metal pipelines are still the main materials of drinking water distribution systems. The percentage of cast iron pipes and steel pipes in drinking water distribution systems amounted to 90% in China, 56.6% in the United States, and 67.2% in Italy [11,12]. Unlined cast iron pipes are still widely used in current distribution systems all over the world, particularly in some old and large cities. Internal corrosion of metal pipes is a common problem in drinking water distribution systems.

**Figure 1:**
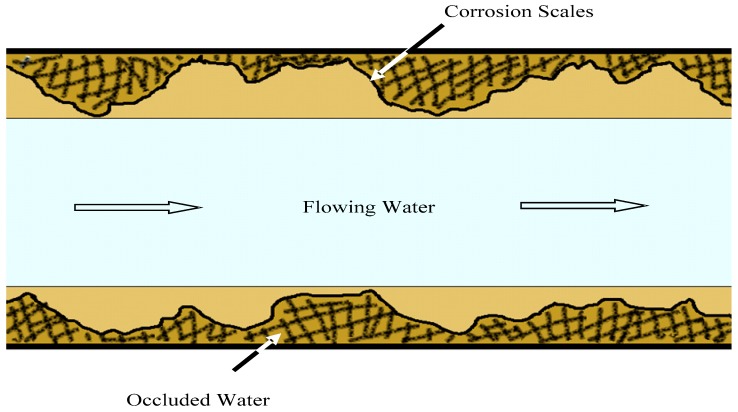
The idea of occluded water and flowing water in the corroded distribution pipe.

Typical iron corrosion scales have a porous structure and are basically composed of three layers: (1) a surface layer, (2) a hard, shell-like layer, and (3) a porous core layer [13,14]. Sarin’s research shows that the porosity of corrosion scales is 40–55% [13]. The porous core contains particles and liquids. On the basis of previous studies [15,16,17], we have carried out an in-depth analysis of the occluded water—the water that remains in the interior of corrosion scales. The occluded water is illustrated in Figure 1 [18]. We found that the quality of occluded water is very different from that of flowing water. Occluded water is weakly acidic and contains a lot of ions such as iron, manganese, chlorine and sulfate [18]. However, due to the lack of research on occluded water, it is still unknown whether other metal ions such as copper and chromium are enriched in occluded water, what the main source of the high concentrations of iron and manganese is, and how the occluded water affects the quality of flowing water.

Heavy metal pollution in drinking water has become a growing problem worldwide. Although some heavy metals are necessary for normal body growth and functions of living organisms, some metals like copper are considered highly toxic when present in excessive amounts. Some other metals such as chromium may even be toxic to humans at very low concentrations [19,20]. The heavy metals—copper and chromium—both exist in ductile iron and corrosion scales, and in addition to iron and manganese, the contents of copper and chromium are relatively high in ductile iron. Therefore, based on previous studies [13,14,15,16,17,18], the current study focused on the corrosion of pipes caused by occluded water, and applied an occluded corrosion cavity simulation cell to analyze changes in the concentrations of metal ions (total copper, total chromium, total iron, total manganese) in occluded water. The study also investigated whether the metal ions in occluded water were released into the flowing water and explored the sources of high concentrations of iron and manganese in occluded water.

## 2. Materials and Methods

### 2.1. Experimental Set Up

**Figure 2:**
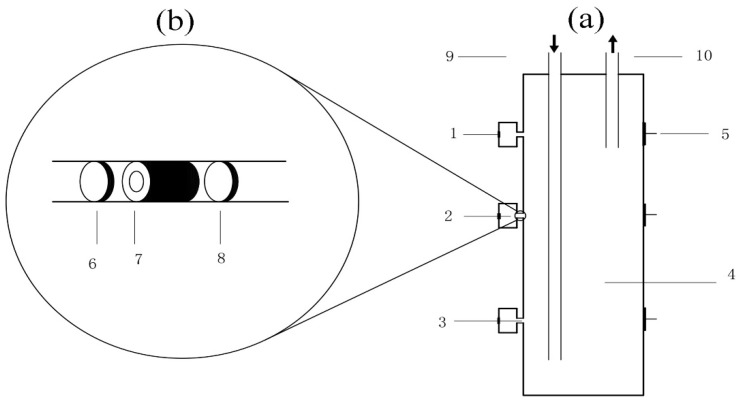
A schematic diagram of the occluded corrosion cavity simulation cell (**a**) and the enlarged schematic diagram of the diffusion channel in the occluded corrosion cavity simulation cell (**b**). (1) anode, (2) occluded area, (3) diffusion channel, (4) flowing area, (5) cathode, (6) filter paper, (7) simulation corrosion scales, (8) filter paper, (9) flowing water inlet, (10) flowing water outlet.

Referring to previous research [18,20,21], an occluded corrosion cavity simulation cell was designed and used in this study (Figure 2a,b). The volumes of the occluded area and the flowing area were 2 mL and 2000 mL, respectively. The simulation corrosion scales were filled in the diffusion channel. In order to study the sources of iron, manganese, copper, and chromium in the occluded water, and to study the role of corrosion scales in the migration of these metals, two kinds of simulation corrosion scales were used in the experiment. One of the simulation corrosion scales (SCS I) was a mixture of Fe_2_O_3_, Fe_3_O_4_, and Fe(OH)_3_ in the proportion of 13:7:2 [21]. Filter paper pieces were used as other simulation corrosion scales (SCS II). In the experiment, the anode electrode material was ductile iron (Table 1). Before the experiment, the anode surface was polished with emery paper from 360 grit to 2000 grit, and then was cleaned with ultra-pure water, degreased by anhydrous ethanol, and finally dried. In order to avoid the interference of cathode corrosion, a graphite electrode was used as the cathode. The device was made of polytetrafluoroethylene to avoid the effects of the device’s materials on the experiment.

### 2.2. Experimental Operation

#### 2.2.1. Phase I: The Change in Metal Concentration in Occluded Area

The experiment unit operated at a flow velocity of 400 mL/min and current of 0.1 mA [22], and the simulation water (SW1) was added in both the flowing area and the occluded area. The main water quality parameters of SW1 are shown in Table 2. The anions (Cl^-^, SO_4_^2-^, and NO_3_^-^) in SW1 were adjusted by NaCl, Na_2_SO_4_, and NaNO_3_, respectively. According to the upper limit of drinking water quality standard, Fe, Mn, Cu, Cr, Ca, and Mg ions in SW1 were adjusted by FeSO_4_, MnSO_4_, CuCl_2_, CrCl_3_, CaCl_2_, and MgCl_2_, respectively. In this experiment, SCS I was used. The occluded water was sampled on the 4th, 8th, 12th, 20th, 28th, and 36th day, respectively. The concentrations of total iron, total manganese, total copper, and total chromium in the occluded water was measured by inductively coupled plasma mass spectrometry (ICP-MS). The flowing water was replaced with SW1 every two days during the whole experiment. The experiment was conducted in duplicate to ensure the reliability of the results. Mean concentrations of metal ions and standard deviation were calculated.

#### 2.2.2. Phase II: The Source and Migration of Metals in the Occluded Water

The experiment was conducted with the same conditions as in Phase I. The flowing area was filled with the simulation flowing water (SFW2). The ion concentration of Fe, Mn, Cu, and Cr in SFW2 was not adjusted. The occluded area was filled with the simulation occluded water (SOW3). The SOW3 was deoxygenated by nitrogen for 2 hours. The concentrations of iron and manganese in the SOW3 were designed based on the concentrations measured in the previous research [18], while the concentrations of copper and chromium were designed according to the upper limit of the drinking water quality standard of China [23]. The main water quality parameters of SFW2 and SOW3 are shown in Table 2. The SCS I and SCS II were filled in the diffusion channel respectively. According to the experimental results in Phase I, it was found that the change in metal ion concentration could be observed within 60 hours. In order to reduce the study time, this test period was set at 60 hours, and water samples from the occluded area and the flowing area were respectively collected at the 12th, 24th, 36th, 48th, and 60th hour. The concentrations of total iron, total manganese, total copper, and total chromium in the flowing water were measured by ICP-MS. The experiment was conducted in duplicate to ensure the reliability of the results. Mean concentrations of metal ions and standard deviation were calculated.

### 2.3. Analysis Method

Each sample was divided into two parts. One part was filtered through a 0.45 μm membrane, and the other part was not filtered. The filtered samples were used for the analysis of chlorine, nitrate, and sulfate ions. The unfiltered samples were acidified with 2% nitric acid and analyzed for the metal concentrations [24]. Water samples were measured for pH, conductivity, and dissolved oxygen (DO). These parameters were measured using a METTLER TOLEDO multi-parameter instrument. Inorganic anions were determined by ion chromatography using a DIONEX DX-600 instrument. Metal ions were determined by ICP-MS (Agilent 7900). Instruments were calibrated using mixed, acidified standards, and internal standardization during ICP-MS analysis was achieved by the addition of 100 μg mL^−1^ of Bi, Ge, In, Li-6, Lu, Rh, Sc, and Tb. The operating conditions of ICP-MS were as follows: carrier gas: 1.05 L min^−1^; radio-frequency (RF) power: 1500 W. In the experiment of phase II, the metal content in the scales (SCS I) before and after operation were semi-quantitatively determined via the method proposed by the United States Environmental Protection Agency for heavy metals in soils, sediments, and sludge [25]. Briefly, 1 g (dry weight) of crushed scales was digested with repeated additions of nitric acid and hydrogen peroxide. During reagent addition, the sample was heated just below its boiling point for 4 h. After a cooling period, the sample was filtered through a filter paper. The concentrations of metals in the digested samples were analyzed with ICP-MS. The compositions of ductile iron and corrosion scales before the experiment were determined by energy dispersive X-ray spectroscopy.

## 3. Results and Discussion

### 3.1. Study on the Change of Metal Concentration in Occluded Water

**Figure 3:**
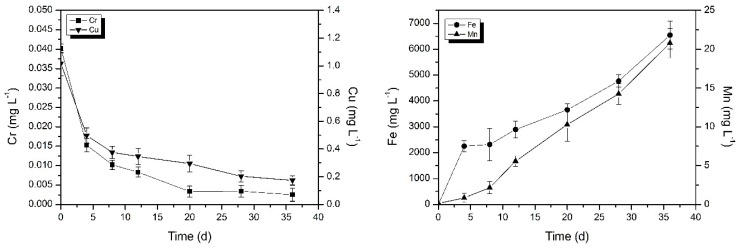
Change in copper, chromium, iron, and manganese mean concentrations in occluded water. Error bars indicate ± standard deviation.

From previous studies [16,18] we can see that iron and manganese are enriched in the occluded water. However, the change process of metal concentration in the occluded water is still unknown, and it is also unknown whether other metal ions will also be enriched in the occluded water. Therefore, we further studied the change of metal concentration in occluded water to get a better idea of the occluded water. It can be seen from Figure 3 that the concentration of copper of the occluded water at the beginning was 1.018 mg L^−1^ and the concentration of chromium was 0.040 mg L^−1^. Thirty-six days later the concentrations of copper and chromium ions in the occluded water decreased by 84.9% and 93.5%, respectively. One of the reasons for this phenomenon may be that corrosion scales are mainly composed of iron oxide (hydroxide) and have an adsorption effect [26,27]. Copper and chromium in the occluded water were adsorbed on the corrosion scales, resulting in a gradual decrease in the concentrations of copper and chromium (Figure 3). In addition, in the aqueous environment, chromium typically exists in either trivalent or hexavalent forms. The trivalent form is relatively insoluble and immobile due to its precipitation as Cr(OH)_3_ [28]. The hexavalent form has a sorption profile, and lower pH values typically enhance its adsorption [28]. So, low pH values in occluded water promote adsorption of hexavalent chromium onto the scales. In previous studies [8,28], the accumulation of metal contaminants in the corrosion scales in the drinking water distribution systems was confirmed. It was reported that high concentrations of copper, cobalt, and nickel were detected in the drinking water distribution systems [29]. On the other hand, as shown in Table 1, the weight percentage of copper and chromium in the ductile iron of the pipe was only 0.3% and 0.16%, respectively. In the same water environment, relative to the iron, copper and chromium were more stable in the pipe and did not undergo redox reaction. Therefore no dissolved copper and chromium entered the occluded water from the pipe. Therefore, the concentrations of copper and chromium in the occluded water did not increase.

The concentration of iron and manganese in the occluded water gradually increased. At the end of the experiment, the concentration of iron and manganese reached 6543.334 mg L^−1^ and 20.784 mg L^−1^, respectively (Figure 3). Nawrocki et al. also found high concentrations of iron and manganese in steady water [16]. This was consistent with the fact that the real occluded water measured previously was rich in high concentrations of iron and manganese [18]. The possible reason for this phenomenon is related to the pipe material and corrosion scales. Gonzalez et al. indicated that iron, manganese, and other metals in drinking water leached from the pipe material and corrosion scales [30]. This phenomenon was also confirmed by the source analysis experiment in the current study.

### 3.2. The Source and Migration of Metal in the Occluded Water

Since the concentrations of iron and manganese in the occluded water are much higher than the drinking water quality standard of China [23], the pollution problem of the drinking water is probably caused by the migration and transformation of metal among occluded water, the corrosion scales, and flowing water. The source of the high concentrations of iron and manganese in the occluded water is still unknown; therefore, this experiment studied the source and migration of metal in the occluded water.

**Figure 4:**
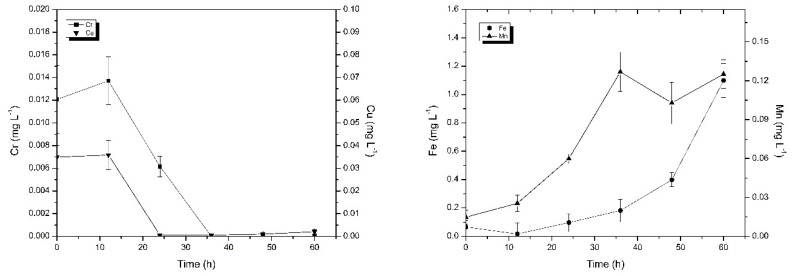
Change in copper, chromium, iron, and manganese mean concentrations in flowing water. Error bars indicate ± standard deviation.

In this experiment the change of copper and chromium concentrations in the flowing water using the SCS I is shown in Figure 4. Initially, the concentrations of copper ions and chromium ions in flowing water were 0.035 mg L^−1^ and 0.012 mg L^−1^, respectively. The concentrations of copper and chromium were firstly increased and then decreased. Finally, the concentrations of copper and chromium were both lower than the detection limit. One of the reasons for the increase of copper and chromium concentrations in the initial stage of the experiment may be the changes in the system hydraulics. At the beginning of the experiment, the flowing water changed from a stagnant state to a flowing state, and some particles of SCS I released into the flowing water under the hydraulic disturbance conditions [31]. The SCS I contained copper and chromium (Table 3). Thus, the concentrations of copper and chromium in the flowing water increased. With the extension of the experiment time, the particles gradually adsorbed on the pipe wall, which was also the reason for the decrease of copper and chromium concentrations in the flowing water. At the end of the experiment, the concentrations of copper and chromium in the occluded water both decreased. The concentration of copper decreased from 1.015 mg L^−1^ to 0.379 mg L^−1^, and the concentration of chromium decreased from 0.059 mg L^−1^ to below the detection limit. This illustrated that copper and chromium in the occluded water migrated outwards. The results of the concentrations of metal elements in SCS I before and after this experiment are shown in Table 4. It shows that the concentrations of copper and chromium in SCS I after operation increased. This also illustrates that copper and chromium in the occluded water migrate outwards and adsorb onto the scales during the migration process. Moreover, the scales also adsorb copper and chromium in the flowing water, resulting in an increase in copper and chromium concentration in the corrosion scales. This is also one of the reasons for the decrease in the concentrations of copper and chromium in the flowing water.

In this experiment, changes in the concentrations of iron and manganese in the flowing water using the SCS I are shown in Figure 4. It can be seen from Figure 4 that the concentrations of iron and manganese in flowing water were increased over time. The manganese concentration in the flowing water reached 0.125 mg L^−1^ at the end of the experiment, which was significantly increased compared to the initial manganese concentration (0.014 mg L^−1^). In addition, the concentration of manganese in the occluded water increased from 17.311 mg L^−1^ to 17.957 mg L^−1^, and the manganese content in the scales also increased. This indicates that the manganese in the pipe also participates in the corrosion reaction, resulting in dissolved manganese entering into the occluded water, and a portion of the manganese in the occluded water adsorbed onto the SCS I. Due to the autocatalytic theory [32] of corrosion occluded cells, under the effect of the electric field, a portion of the manganese in the occluded water migrated to the flowing water, causing concentrations of manganese in the flowing water to increase. Similarly, the concentration of iron in the occluded water (from 934.214 mg L^−1^ to 3340.361 mg L^−1^) and the concentration of iron in the flowing water (from 0.066 mg L^−1^ to 1.099 mg L^−1^) both increased, indicating that the corrosion of the pipe causes a large amount of dissolved iron to enter into the occluded water, and a part of iron in the occluded water is also released into the flowing water, resulting in an increase in the concentration of iron in the flowing water. The reason for the decrease of manganese concentration in the later stage may be that Mn(II) could be oxidized into particulate forms much easier [33] and with the stability of hydraulic conditions, manganese oxides adsorbed on the pipe wall or corrosion scales.

**Figure 5:**
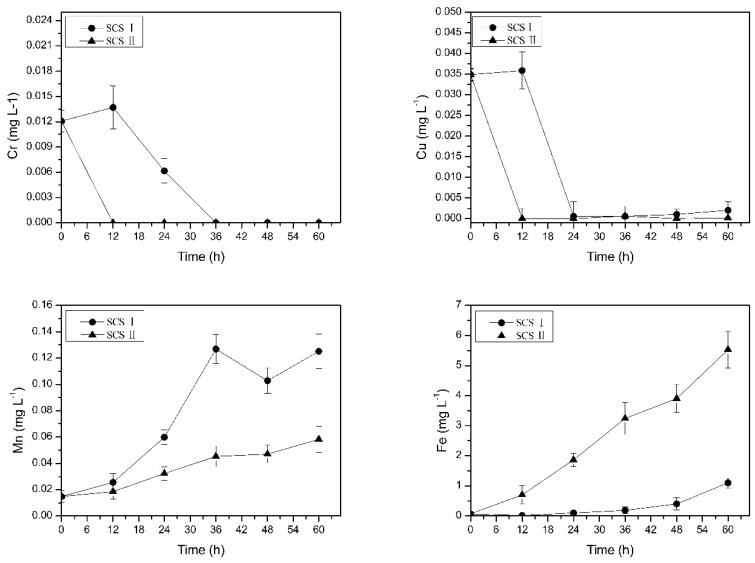
Change in mean concentrations of metal ions in flowing water under two different simulated corrosion scales conditions. Error bars indicate ± standard deviation.

**Figure 6:**
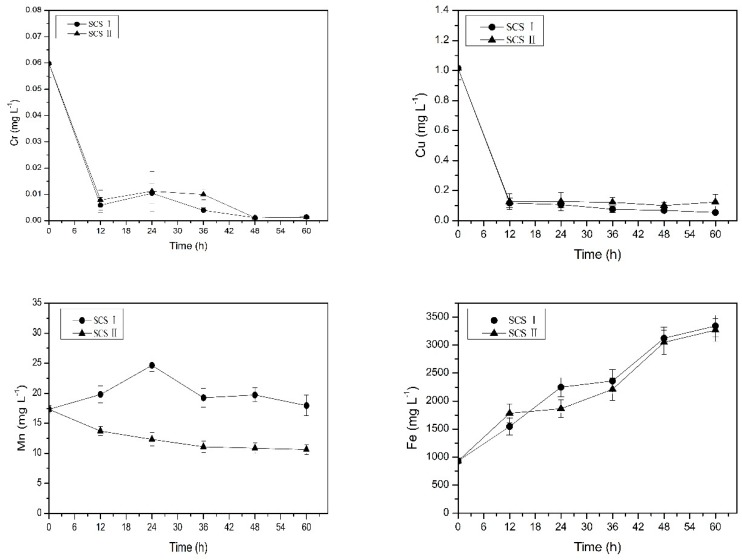
Change in mean concentrations of metal ions in occluded water under two different simulated corrosion scales conditions. Error bars indicate ± standard deviation.

The above experiments show that corrosion of the pipe is one of the sources of high concentration of iron and manganese in the occluded water. To further analyze its source and explore the role of pipe scales, we used SCS II instead of SCS I for further experiments. The results of the change in the concentrations of copper, chromium, manganese, and iron in the flowing water and in the occluded water are shown in Figure 5 and Figure 6, respectively. The concentrations of copper and chromium in the occluded water and flowing water both continuously decreased. At the end of the experiment, the occluded water and flowing water almost did not contain copper and chromium. This illustrated that they adsorbed on the pipe wall or the simulated scales. When there were changes in the system hydraulics, copper and chromium would release into flowing water again and caused the pollution of the flowing water [31].

Under two different simulated corrosion scales conditions, the changes of iron in the occluded water were almost the same and both increased with time. This further illustrates that high concentrations of iron in the occluded water derived from corrosion of the pipes. The concentrations of iron in the flowing water under two different simulated corrosion scales conditions both increased with time, but the concentrations of iron in the flowing water using the SCS II was higher than that using the SCS I. This indicates that the iron migrates from the occluded water to the flowing water cause the pollution of the flowing water, and the increase of iron in the flowing water mainly comes from the occluded water. The existence of SCS I prevented the migration of iron from the occluded water to the flowing water, whereas the SCS II had little hindrance to their migration. The iron content in the SCS I reduced (Table 4). The pH affected the precipitation and dissolution of iron. The acidic environment of the occluded water caused the dissolution of iron oxide and hydroxide in the scales.

In the SCS II group, the manganese concentration increased in the flowing water but decreased in the occluded water. This shows that manganese migrates from the occluded water to the flowing water. Manganese in the pipe participates in the corrosion reaction, which produces soluble manganese into the occluded water, but the concentration of manganese in the occluded water will decrease; this indicates that the migration rate of manganese in the SCS II group is greater than its dissolution rate, and the SCS II has less hindrance to the migration. However, the manganese concentration in the flowing water using the SCS I was higher than that using the SCS II (Figure 5). In the SCS I group the manganese concentration in the occluded water (Figure 6) and the manganese content in the SCS I (Table 4) both increased. This indicates that the SCS I also had less hindrance to the migration, and would absorb manganese, but not as much as the SCS II. Manganese in flowing water is mainly from the metal pipe corrosion in the occluded area. Manganese oxides are often present in fine-grained mixtures that do not have good crystallinity [34], which is different from the iron that may form a hard crystalline corrosion scales [35]. Therefore, manganese is more labile to be released under hydraulic disturbance [33]. Thus, not only manganese migrates from occluded water to flowing water, but also the manganese in SCS I releases into flowing water in certain situations.

## 4. Conclusions

An anodic reaction occurs in the cast iron pipe wall, releasing a large amount of iron and manganese ions. This is the main source of the high concentration of iron and manganese in the occluded water. Some of the iron and manganese ions migrate into the flowing water causing water quality pollution. However, due to the inhibition of the scales, the outward migration rate of iron and manganese is less than the corrosion rate, so the occluded water is enriched with a large amount of iron and manganese. Manganese in occluded water and flowing water also can absorb onto the corrosion scales. Owing to the adsorption and co-precipitation of metal oxides in the scales, copper and chromium ions in the flowing water and occluded water also can adsorb on the scales. Manganese, copper, and chromium can release into the flowing water again under hydraulic disturbance. The metal oxides in the scales may be dissolved into the occluded water under the action of hydrogen ions. The results of this study further deepen peoples’ understanding of occluded water, which is the theoretical basis for controlling pollution of drinking water in the future.

## Figures and Tables

**Table 1:** Elemental composition of ductile iron by energy dispersive X-ray spectroscopy.

Element	Weight Percentage (%)	Atomic Percentage (%)	Strength
C	3.58	14.63	10.20
Na	0.18	0.37	0.99
Mg	0.19	0.38	2.18
Al	0.16	0.28	2.57
Si	0.04	0.06	0.83
Sr	0.04	0.02	0.49
Pb	0.10	0.02	2.15
Ca	0.15	0.19	3.64
Cr	0.16	0.15	3.42
Mn	0.70	0.54	4.27
Fe	94.42	83.07	1017.64
Cu	0.30	0.26	4.07

**Table 2:** The main water quality parameters of SW1, SFW2, and SOW3.

	SW1	SFW2	SOW3
pH	7.06	7.03	3.95
DO (mg L^−1^)	7.50	7.96	<0.50
Cond (us cm^−1^)	544.00	549.70	4023.80
Cl^-^ (mg L^−1^)	62.56	69.31	594.72
SO_4_^2-^ (mg L^−1^)	143.43	152.62	1481.13
NO_3_^-^ (mg L^−1^)	13.23	10.18	4.98
Fe (mg L^−1^)	0.384	0.065	934.210
Mn (mg L^−1^)	0.130	0.014	17.310
Cu (mg L^−1^)	1.018	0.035	1.015
Cr (mg L^−1^)	0.040	0.012	0.059
Ca (mg L^−1^)	4.264	4.267	17.012
Mg (mg L^−1^)	20.453	22.122	16.751

SW1: simulation water			
SFW2: simulation flowing water			
SOW3: simulation occluded water DO: dissolved oxygen			

**Table 3:** Elemental composition of simulation corrosion scales (SCS) I by energy dispersive X-ray spectroscopy.

Element	Weight Percentage (%)	Atomic Percentage (%)	Strength
C	10.09	33.13	14.42
Na	1.64	2.83	5.24
Mg	0.46	0.75	2.97
Si	3.41	4.79	38.26
Sr	1.30	0.59	7.33
Pb	1.66	0.31	12.27
Ca	0.84	0.84	7.92
Cr	0.30	0.21	2.07
Mn	0.16	0.10	0.85
Fe	78.98	55.74	347.50
Cu	1.16	0.71	3.02

**Table 4:** SCS I composition before and after experiment.

	Mg (ug g^−1^)	Al (ug g^−1^)	Mn (ug g^−1^)	Cu (ug g^−1^)	Cr (ug g^−1^)	Ni (ug g^−1^)	Fe (ug g^−1^)	As (ug g^−1^)	Zn (ug g^−1^)
Before	9.521	38.100	32.054	8.296	3.696	2.769	85.401	12.925	20.028
After	5.909	57.474	39.857	11.433	9.361	3.815	69.634	16.509	34.086
